# Vitronectin promotes proliferation and metastasis of cervical cancer cells via the epithelial-mesenchymal transition

**DOI:** 10.3389/fonc.2024.1466264

**Published:** 2024-12-09

**Authors:** Yao Lin, Lihong Bian, Guangwei Zhu, Bin Zhang

**Affiliations:** ^1^ Department of Gynecology and Obstetrics, the First Affiliated Hospital, Fujian Medical University, Fuzhou, China; ^2^ Department of Gynecology and Obstetrics, National Regional Medical Center, Binhai Campus of the First Affiliated Hospital, Fujian Medical University, Fuzhou, China; ^3^ Department of Gastrointestinal Surgery 2 Section, the First Affiliated Hospital, Fujian Medical University, Fuzhou, China

**Keywords:** vitronectin, cervical cancer, proliferation, metastasis, epithelial-mesenchymal transition (EMT)

## Abstract

**Background:**

Vitronectin (VTN) is a multifunctional glycoprotein in blood and the extracellular, which could be an effective biomarker for many cancers. However, its role in cervical cancer is under investigated. In this study, we aimed to determine the molecular function of VTN and its potential mechanism in cervical cancer (CC).

**Materials and methods:**

Up- and down-regulated VTN expression was determined in Hela and C33A cells. Reverse transcription, qRT-PCR, and Western blotting test were performed to identify VTN mRNA and protein levels, separately. CCK-8 assay and colony formation assay were carried out to evaluate proliferation abilities of CC cells. A scratch test and a transwell chamber assay were performed to determine cell migration and invasion ability. Expression levels of epithelial-mesenchymal transition (EMT)-related proteins were measured by Western blotting.

**Results:**

Cell models with up- and down-regulated VTN expression in Hela and C33A cells were successfully established, as confirmed by Western blotting and qPCR. CCK-8 and colony formation assays demonstrated that VTN overexpression significantly enhanced the proliferation of both Hela and C33A cells. Wound healing and Transwell migration assays further indicated that VTN overexpression markedly promoted the migratory and invasive capabilities of these cells. Moreover, Western blotting analysis revealed that VTN overexpression led to a decrease in ZO-1 and E-cadherin protein levels and an increase in β-catenin and N-cadherin levels, whereas VTN knockdown yielded the opposite effect. These findings suggest that VTN promotes cervical cancer cell malignancy through epithelial-mesenchymal transition (EMT).

**Conclusion:**

VTN plays a tumor-promoting role in CC by promoting the EMT of cervical cancer cells.

## Introduction

Cervical cancer (CC) is a prevalent gynecological malignancy globally, significantly jeopardizing women’s health and well-being (1). Annually, there are approximately 661,000 new cases and nearly 348,000 CC-related deaths, ranking second in incidence and first in mortality among women’s malignant neoplasms (2, 3). Evidence has demonstrated that the 5-year overall survival rate of early-stage CC patients is about 70%. However, it drops to as low as 30 for patients in advanced stages (4, 5). Surgery is the primary therapeutic approach for CC in the early stage, and radiotherapy is widely chosen for advanced CC according to NCCN guidelines (6, 7). However, even with the incorporation of comprehensive modalities such as radiotherapy, chemotherapy and targeted agents, about 20% of CC patients will still end up with distant metastases such as lung or lymph node metastases, further impairing their survival outcomes (8). Hence, identifying specific prognostic markers for CC recurrence and metastasis holds immense clinical significance for CC management.

Vitronectin (VTN) is a type of secreted glycoprotein composed of 459 amino acids, which participates in the formation of extracellular matrix (ECM) and exhibits high expression in various tumor tissues (9, 10). By interacting with integrins on cell membranes, VTN facilitates cell adhesion, migration, and proliferation, potentially impinging on tumor cell growth, migration, and metastasis within the microenvironment of malignant tumors (11). A large body of literature has proven that VTN is pivotal in cancer malignancy by activating cell invasion and metastasis. For example, Niu et al. demonstrated that VTN expression was highest in metastatic prostate cancer, followed by primary prostate cancer tissue and benign prostatic hyperplasia tissue, suggesting its association with prostate cancer progression and metastasis (12). Zhu et al. also discovered that VTN promoted the malignant behavior of the liver cancer cell line (13). Moreover, VTN and fibronectin in ascites promoted cell adhesion and performed indispensable in peritoneal metastasis of ovarian cancer (14). Nevertheless, as far as we know, no relevant reports exist on the functions and regulatory mechanisms of VTN in CC.

Inorder to explore the function of VTN in CC proliferation and metastasis, the present study conducts over-expression and silencing of VTN in CC cells to evaluate its impact on cell malignancy such as proliferation, migration, and invasion. We hope our study lays the groundwork for further comprehending the mechanisms underlying the onset and progression of CC and provides plausible targets for CC therapy.

## Materials and methods

### Cell cultures

C33A and Hela, types of the human cervical cancer cell lines,were purchased from the American Type Culture Collection (ATCC, Manassas, VA, USA). RPMI-1640 medium which contained 10% fetal bovine serum and 0.1% penicillin-streptomycin was used to culture the CC cells.Cells were maintained in a humidified incubator at 37°C with 5% CO_2_ subsequently.

### Plasmids and transfection

The open reading frame of human VTN was integrated into the eukaryotic expression vector GFP-pCDH (System Biosciences, Inc.). The resulting recombinant plasmid, pCDH-VTN, was co-transfected with lentivirus plasmids psPAX2 and pMD2.G (Genechem Inc, Shanghai, China) into HEK293T cells using Lipofectamine 3.0 (Invitrogen) at a concentration of 60%, according to the manufacturer’s instructions. The cells were cultured for 48 hours after transfection. Subsequently, Hela and C33A cells, following lentivirus transfection, underwent selection with puromycin at a concentration of 1 μg/mL1 μg/mL (Invitrogen) for a period of two weeks.

### Quantitative real-time polymerase chain reaction

Total RNA was extracted from cells using Trizol reagent (Thermo Fisher Scientific Inc.) according to the manufacturer’s instructions. Then, cDNA was synthesized and qRT-PCR was performed using a cDNA Reverse Transcription kit and Step One Real-Time PCR system (Applied Biosystems, CA, USA) in an ABI Prism 7900HT instrument (Applied Biosystems) according to the supplier’s recommendations. The primers used were as follows: VTN: F: 5′-GGGTCTACTTCTTCAAGGGGAA−3′, R: 5′-AATGAACTGGGGCTGTCTGG−3′; GAPDH: F: 5′-GGAGCGAGATCCCTCCAAAA T−3′, R: 5′-GGCTGTTGTCATACTTCTCATGG-3′. All values were normalized to the level of GAPDH. The relative mRNA expression of VTN was calculated using the 2^-ΔΔCt^ comparative method. All experiments were repeated three times.

### Western blot analysis

The cells were disrupted by RIPA buffer fortified with a protease inhibitor concoction and protein measured with a BCA protein assay kit (Beyotime). After that, the cell lysates were fractionated using SDS-PAGE, followed by the transferation of equivalent protein quantities onto polyvinylidene difluoride (PVDF) membranes (Millipore). Following blockage, the PVDF membranes were subjected to overnight incubation at 4°C in the presence of primary antibodies. Detection of the primary antibodies was achieved using secondary goat anti-rabbit or goat anti-mouse HRP-IgG (Proteintech, Rosemont, IL, USA). Subsequently, immunoreactive complexes were visualized via a chemiluminescent substrate and documented on film.

### Cell proliferation assay

Cells were centrifugated,with a concentration of 10^4^ cells/mL, and then were seeded in a 96-well plate with 100 µL of culture medium per well. Each group was established with four replicate wells. The plate was then placed in a 37°C incubator to facilitate continued cultivation. After seeding, the cells were collected, and cell quantification was undertaken using the CCK-8 on days one, two, three, and four. CCK-8 was added to each well and subsequently layed in a 37°C incubator for further two hours. The absorbance was counted with a microplate reader. The experiment was iterated three times.

### Cell colony formation assay

Cells (100 µL) were added to a 6-well plate with a concentration adjusting to 8×10^3^ cells/mL, subsequently cultured for seven days, and the culture medium were refreshed every two days. Crystal violet was used to stain the colonies. For each condition, the number of colonies within a given area was counted. All experiment was repeated three times.

### *In vitro* wound healing cell migration

Cells were seeded at a density of 1×10^^6^ cells per well in a 6-well plate and cultured at 37°C with 5% CO₂ until reaching 90% confluence, forming a uniform monolayer. A sterile 200 μL pipette tip was used to create a straight scratch across the cell layer, taking care to apply even pressure to ensure a consistent width without damaging the plate surface. The cells were then rinsed twice with phosphate-buffered saline (PBS) to remove any detached cells, followed by the addition of serum-free medium. The plates were incubated at 37°C with 5% CO₂, and the wound width was observed under a microscope at 0 and 24 hours.

### Transwell invasion assay

Cells (50 μL) were initially prepared by suspending in serum-free RPMI-1640 medium and added to Transwell chamber inserts to ensure proper density and even distribution. For each experimental group, 200 μL of cell suspension containing 1×10^^6^ cells was carefully transferred into the upper chamber of a Transwell insert, which is designed to allow cell migration through a porous membrane. RPMI-1640 medium containing 20% fetal bovine serum (FBS) was added to the lower chamber, creating a chemoattractant gradient to encourage cell invasion. The Transwell plates were incubated at 37°C with 5% CO₂ for 24 hours to allow cells to migrate toward the serum in the lower chamber. After incubation, the non-invading cells on the upper surface of the membrane were gently removed using a cotton swab. The chambers were then rinsed twice with phosphate-buffered saline (PBS) to remove any residual medium. Invading cells attached to the lower surface of the membrane were fixed with 4% paraformaldehyde for 20 minutes, then stained with 0.1% crystal violet solution for 15 minutes at room temperature. The membrane was thoroughly rinsed to remove excess stain, and the stained cells on the lower surface were visualized and counted under a microscope. The number of invaded cells was recorded for each group, and the experiment was repeated three times for statistical validity.

### Statistical analysis

Statistical analyses were performed using SPSS 21.0 (SPSS, Chicago, Illinois, USA), and the presented values were expressed as mean ± standard deviation (SD). Two-sided t-tests were used for comparisons between two groups, and one-way analysis of variance (ANOVA) was used for comparisons among multiple groups.

## Results

### Identification of VTN expression in cervical cancer cells

**Figure 1:**
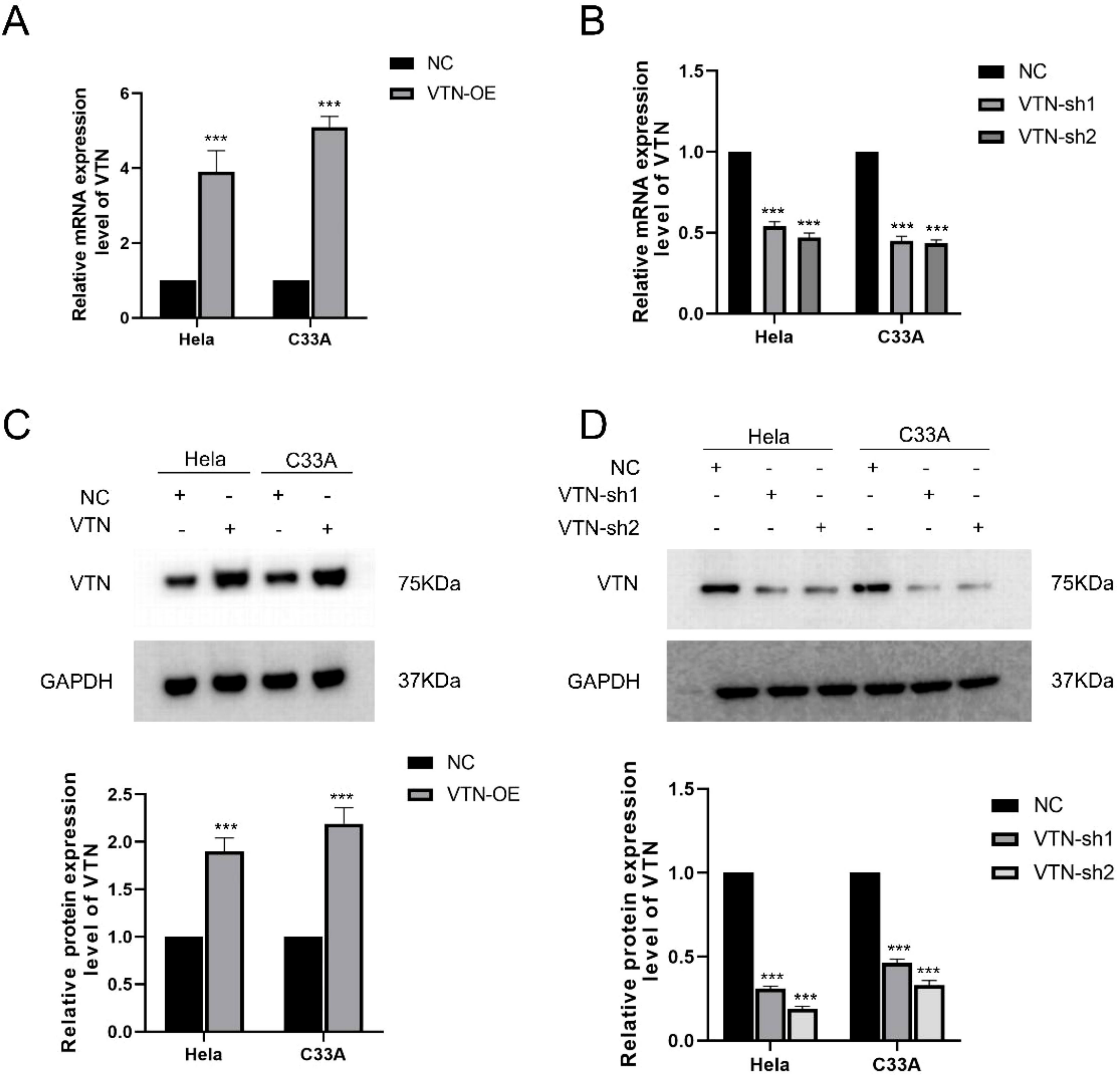
Overexpression and knockdown of VTN in Hela and C33A cells. **(A)** The mRNA levels of VTN were assessed using RT-qPCR with VTN primers in Hela and C33A cells stably transfected with pCDH and pCDH-VTN plasmids. GAPDH was used as an internal control for amplification. Results showed significant elevation of VTN mRNA levels in both cell lines after overexpression of the gene. **(B)** RT-qPCR using VTN primers was performed on total RNA extracted from Hela and C33A cells transfected with NC, shRNA-1, and shRNA-2 sequences. GAPDH was used as an internal control for amplification. The shRNA-1 and shRNA-2 sequences showed inhibition of VTN mRNA expression levels. **(C)** Western blotting was used to detect VTN protein levels in Hela and C33A cells stably transfected with pCDH and pCDH-VTN plasmids. GAPDH protein was used as an internal control. Semi-quantitative analysis results showed that VTN protein levels were significantly higher in the overexpression group than in the NC group, P<0.05. **(D)** Western blotting was used to detect VTN protein levels in Hela and C33A cells transfected with NC, shRNA-1, and shRNA-2 sequences. GAPDH protein was used as an internal control. Semi-quantitative analysis results showed that VTN protein levels were significantly lower in the knockdown group than in the NC group, P<0.05. The mRNA and protein expression of VTN in the NC group of Hela and C33A cells were regarded as 1. Error bars represent mean ± SEM and the results are representative of three experiments. ***P<0.001.

In order to explore the roles of VTN in cervical cancer cells, our group transfected pCDH-VTN to enhance VTN expression and transfected VTN shRNA to inhibit VTN levels in Hela and C33A. Negative controls were established by transfecting cells with scrambled pCDH or shRNA. We quantified mRNA expression using qPCR (**Figures 1A, B**) and protein level using Western blotting (**Figures 1C, D**). The over-expression and down-expression VTN models were successfully built.

### Up-regulated VTN level promoted the proliferation of cervical cancer cells

**Figure 2:**
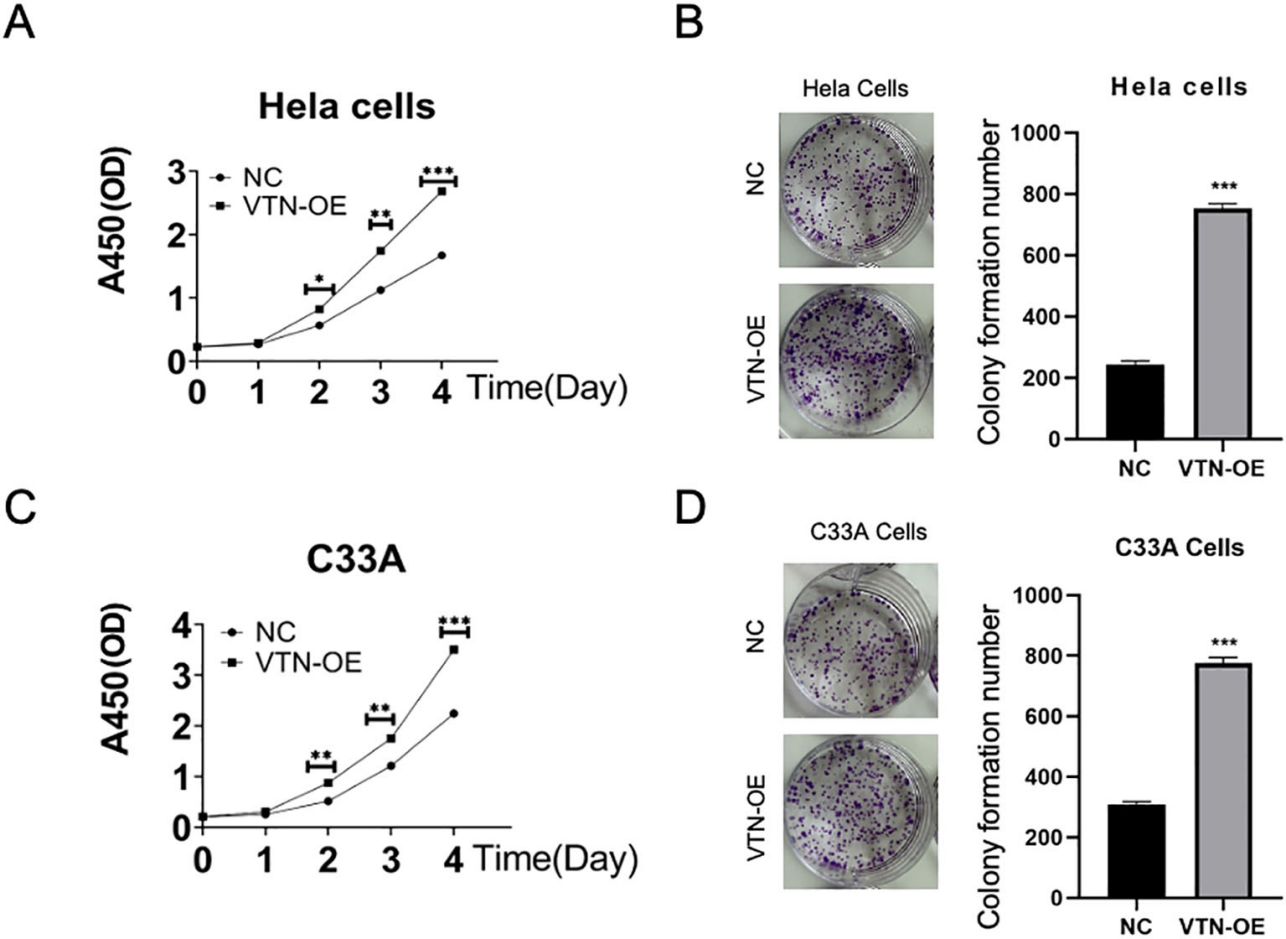
VTN promotes the proliferation of cervical cancer cells. **(A, C)** CCK-8 assay results showed that overexpression of VTN promoted the proliferation of Hela and C33A cells. **(B, D)** Colony formation assay results showed that overexpression of VTN increased the number of colonies formed by Hela and C33A cells. Error bars represent mean ± SEM and the results are representative of three experiments. *P<0.05, **P<0.01, ***P<0.001.

**Figure 3:**
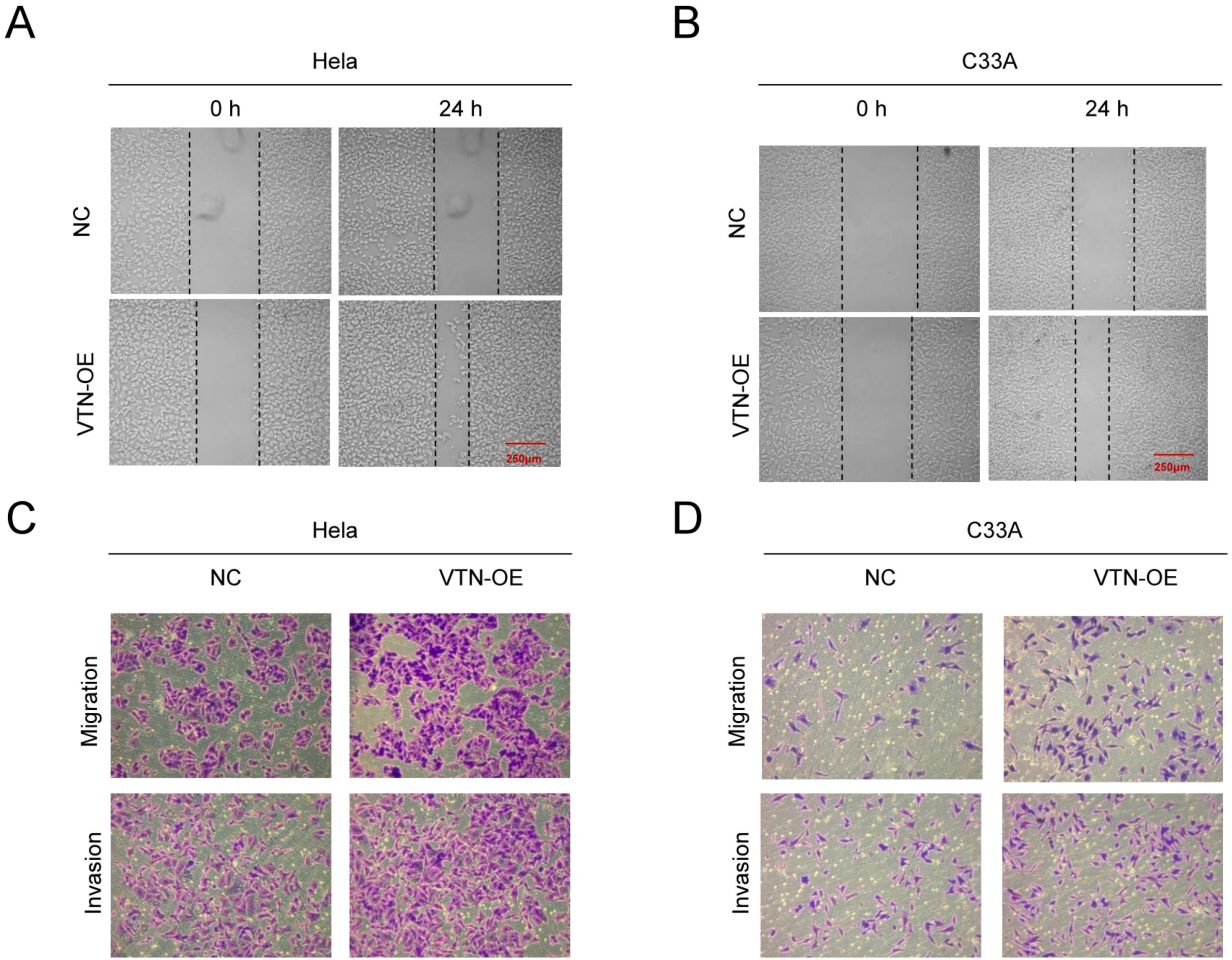
VTN promotes the migration and invasion of cervical cancer cells. **(A, B)** Wound healing assay results showed that overexpression of VTN promoted the migration ability of Hela and C33A cells. **(C, D)** Transwell assay results showed that overexpression of VTN promoted the migration and invasion ability of Hela and C33A cells.

pCDH-VTN transfection in HeLa and C33A promoted cell proliferation (**Figures 2A, C**, *P*<0.001), higher pCDH-VTN transfection colony formation rate was found in both HeLa and C33A cells (**Figures 2B, D**), compared to the control group. Then, pCDH-VTN transfected HeLa and C33A cells resulted in faster wound closure than the control group (**Figures 3A, B**, *P*<0.001). Furthermore, the rate of distance reduction between wound edges was increased in the VTN overexpressing group compared to the negative control group (NC group). Up-regulated VTN significantly enhanced the invasion of HeLa and C33A by the Transwell invasion assay,compared with the control group (**Figures 3C, D**, control, *P*<0.001).

### The epithelial-mesenchymal transition mechanism

**Figure 4:**
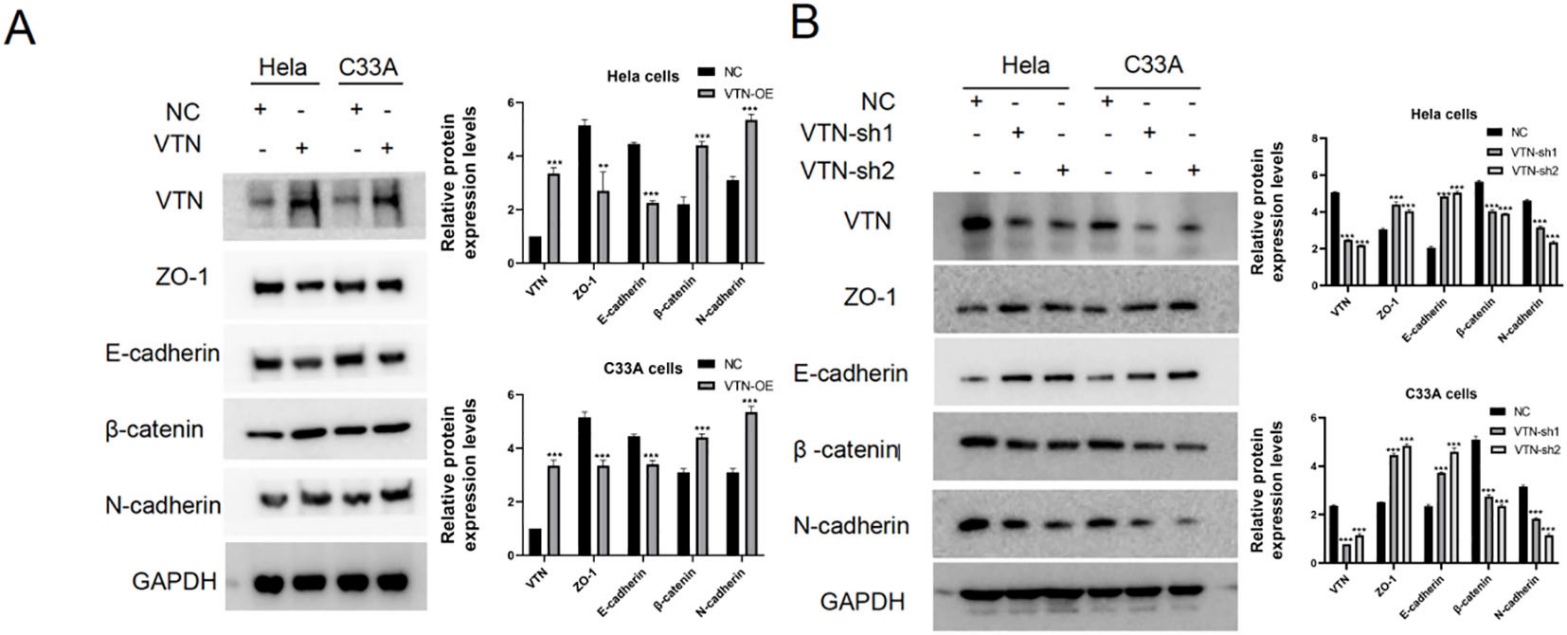
VTN affects the epithelial-mesenchymal transition of cervical cancer cells. **(A)** Western blotting results showed that overexpression of VTN in Hela and C33A cells resulted in decreased protein expression levels of the epithelial-associated proteins E-cadherin and ZO-1, while the mesenchymal-associated proteins N-cadherin and β-catenin were increased. **(B)** Western blotting results showed that knockdown of VTN in Hela and C33A cells resulted in increased protein expression levels of the epithelial-associated proteins E-cadherin and ZO-1, while the mesenchymal-associated proteins N-cadherin and β-catenin were decreased. Error bars represent mean ± SEM and the results are representative of three experiments. **P<0.01, ***P<0.001.

**Figure 5:**
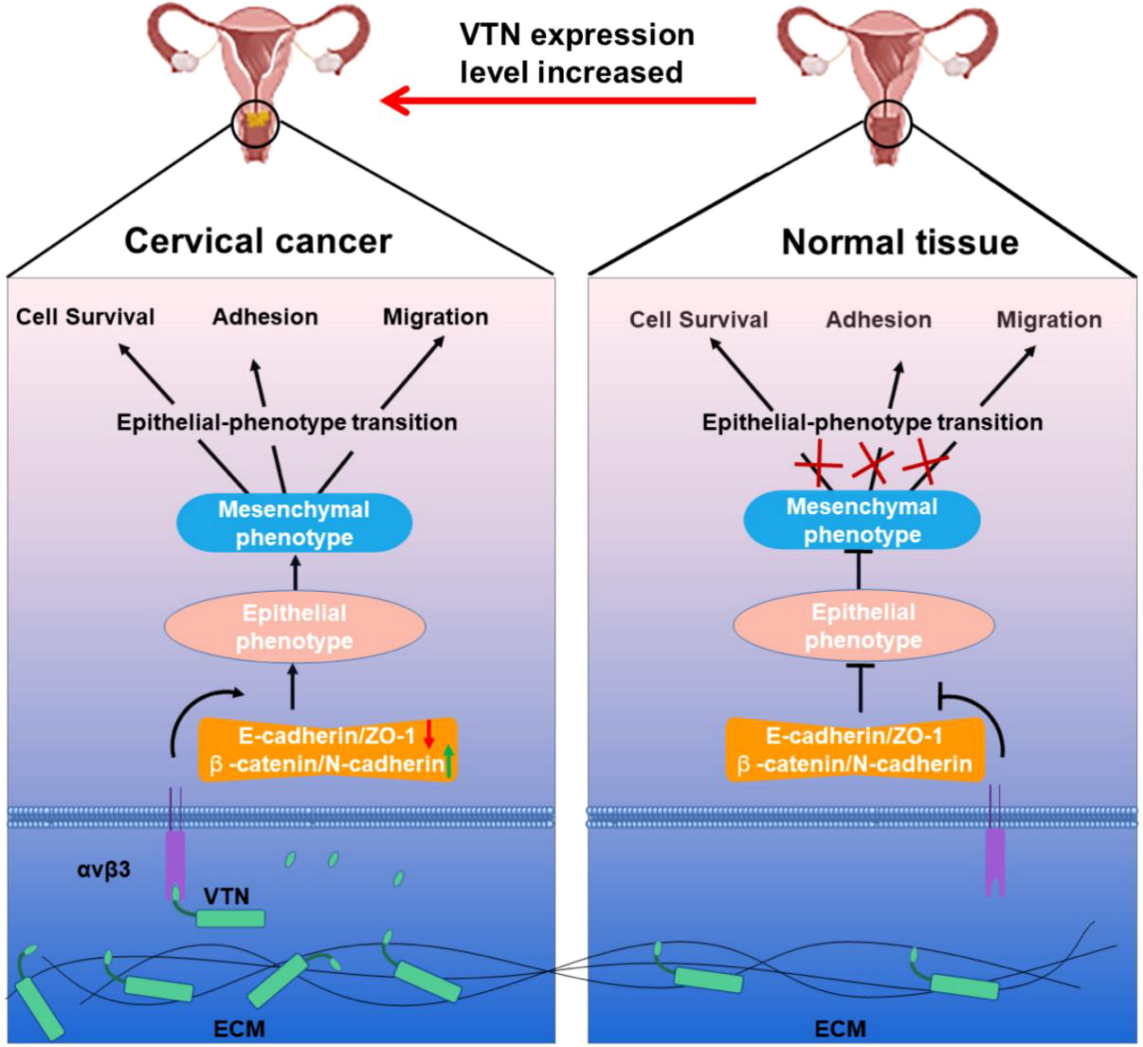
Schematic diagram showing that VTN affects the proliferation, migration, and invasion of cervical cancer cells through EMT.

To investigate the effect of VTN on the epithelial-mesenchymal transition (EMT) of cervical cancer cells, we assessed the expression of typical EMT hallmark proteins, such as ZO-1, E-cadherin, β-catenin, and N-cadherin. Overexpression of VTN in cervical cancer cells led to a decrease in the protein expression levels of ZO-1 and E-cadherin and an increase in the expression levels of β-catenin and N-cadherin (**Figure 4A**). Conversely, the knockdown of VTN expression resulted in increased protein expression levels of E-cadherin and ZO-1, while decreased expression of N-cadherin and β-catenin (**Figure 4B**, P<0.001). These results indicated that VTN played a significant role in regulating the EMT of cervical cancer cells (**Figure 5**).

## Discussion

Our study exhibited that VTN could promote the proliferation, migration, and invasion of CC cells for the first time. We found significant overexpression of VTN in Hela and C33A cells,which promoted cell proliferation, migration, and invasion in CC cells. In addition, down-regulated VTN could inhibit cell biology behavior. We also found that overexpression of VTN reduced the expression of ZO-1 and E-cadherin and upraised the β-catenin and N-cadherin levels. Meantime, down-regulated VTN levels resulted oppositely. Hence, VTN might regulate the proliferation and metastasis of CC cells by switching from E-cadherin to N-cadherin.

Studies showed that VTN was a tumor promoter. From the fundamental perspective, the knockdown of VTN could significantly inhibit the proliferation in cancer cells of liver and the tumor size in xenografts (15). Up-regulated VTN expression could stimulate ovarian cancer cell migration (16). In addition, VTN could promote tumor cell growth in a concentration-dependent manner (13). From clinical research, studies have shown that there was significantly higher level of serum VTN in breast cancer patients with early stage than those in healthy people, patients with benign breast lesions or pre-cancerous lesions, and advanced breast cancer patients. Size of tumor, lymph node status, and clinical stage were negatively correlated the the serum VTN level (17). Vitronectin concentration levels were involved and connected to the tumor recurrence and metastasis of breast cancer (18). Consistent with the above studies, we found that VTN was a tumor promoter gene in CC. Therefore, as an essential tumor promoter gene, treatment targeting VTN could be a therapeutic target.

The biomolecular mechanism by which VTN contributes the oncology potential of CC cells has yet to be determined. In this study, we found that overexpression of VTN decreased ZO-1 and E-cadherin level and enhanced β-catenin and N-cadherin protein expression. Meantime, knocking down VTN expression produced the opposite results. Hence, we deduced that VTN might promote cervical cancer by EMT. An arginine-glycine-aspartic acid (RGD) peptide sequence exists in VTN, which acts as the ligand for αvβ3 integrin on the surface of vascular endothelial cells (19). The specific binding between RGD and integrin mediates signal transduction between cells and ECM, ultimately engendering tumor cell migration (20). In addition, VTN could also function in other signaling pathways. Previous studies have found that VTN could also activate LIF and IL-6 through the integrin-FAK and uPAR signaling pathways to exert functional effects (19). Zheng et al. reported that the cell migration function of VTN was mainly involved in the PI3K/AKT pathway (21). A scholar recently found that VTN could induce tumor cell proliferation through the JNK and ERK pathways (22). Multiple studies have also shown that PI3K/AKT pathway and ERK signaling pathway can impact the function of tumor cells by affecting their EMT (23–26). These findings suggest that VTN could regulate the EMT of cells through multiple signaling pathways. More experimentations are still needed to verify this hypothesis shortly.

Despite the achievements of the current researches, our study has several limitations that should be solved with further researches. First, the mechanism of action of VTN in tumors is still not fully understood, and its impact on tumor progress is complex and may be regulated by multiple factors. Second, cell proliferation was not presented in the other cervical cancer cell lines except for C33A and Hela and the current experiments were performed *in vitro*; thus, *in vivo* experiments are furtherly required to support the present results. Third, further in-depth study of the mechanism of action of VTN in the cervical tumor microenvironment, especially its interaction network with other extracellular matrix proteins, growth factors, and signaling pathways are warranted to illustrate the potential regulatory mechanism.

## Conclusion

VTN can promote tumor growth and metastasis by regulating the EMT process. VTN might be a potential prognostic marker and meaningful new therapeutic target for cervical cancer.

## Data Availability

The original contributions presented in the study are included in the article/supplementary material. Further inquiries can be directed to the corresponding author.

## References

[1] Voelker RA. Cervical cancer screening. JAMA. (2023) 330:2030. doi: 10.1001/jama.2023.21987 37889510

[2] Cohen PA Jhingran A Oaknin A Denny L. Cervical cancer. Lancet. (2019) 393:169–82. doi: 10.1016/S0140-6736(18)32470-X 30638582

[3] Bray F Laversanne M Sung H Ferlay J Siegel RL Soerjomataram I. Global cancer statistics 2022: GLOBOCAN estimates of incidence and mortality worldwide for 36 cancers in 185 countries. CA Cancer J Clin. (2024) 74:229–63. doi: 10.3322/caac.21834 38572751

[4] Matsuo K Machida H Mandelbaum RS Konishi I Mikami M. Validation of the 2018 FIGO cervical cancer staging system. Gynecol Oncol. (2019) 152:87–93. doi: 10.1016/j.ygyno.2018.10.026 30389105 PMC7528458

[5] Sengayi-Muchengeti M Joko-Fru WY Miranda-Filho A Egue M Akele-Akpo M-T N'da G. Cervical cancer survival in sub-Saharan Africa by age, stage at diagnosis and Human Development Index: A population-based registry study. Int J Cancer. (2020) 147:3037–48. doi: 10.1002/ijc.33120 32449157

[6] Abu-Rustum NR Yashar CM Arend R Barber E Bradley K Brooks R. NCCN guidelines® Insights: cervical cancer, version 1.2024. J Natl Compr Canc Netw. (2023) 21:1224–33. doi: 10.6004/jnccn.2023.0062 38081139

[7] Liontos M Kyriazoglou A Dimitriadis I Dimopoulos M-A Bamias A. Systemic therapy in cervical cancer: 30 years in review. Crit Rev Oncol Hematol. (2019) 137:9–17. doi: 10.1016/j.critrevonc.2019.02.009 31014518

[8] Colturato LF Signorini Filho RC Fernandes RC Gebrim LH Oliani AH. Lymph node micrometastases in initial stage cervical cancer and tumoral recurrence. Int J Gynaecol Obstet. (2016) 133:69–75. doi: 10.1016/j.ijgo.2015.08.019 26868069

[9] Biasella F Plössl K Karl C Weber BH Friedrich U. Altered protein function caused by AMD-associated variant rs704 links vitronectin to disease pathology. Invest Ophthalmol Vis Sci. (2020) 61:2. doi: 10.1167/iovs.61.14.2 PMC7718807 33259607

[10] Martí-Pàmies I Cañes L Alonso J Rodríguez C Martínez-González J. The nuclear receptor NOR-1/NR4A3 regulates the multifunctional glycoprote*in vitro*nectin in human vascular smooth muscle cells. FASEB J. (2017) 31:4588–99. doi: 10.1096/fj.201700136RR 28666984

[11] Ciregia F Deroyer C Cobraiville G Plener Z Malaise O Gillet P. Modulation of α(V)β(6) integrin in osteoarthritis-related synovitis and the interaction with VTN((381-397 a.a.)) competing for TGF-β1 activation. Exp Mol Med. (2021) 53:210–22. doi: 10.1038/s12276-021-00558-2 PMC8080589 33526813

[12] Niu Y Zhang L Bi X Yuan S Chen P. Evaluation of vitronectin expression in prostate cancer and the clinical significance of the association of vitronectin expression with prostate specific antigen in detecting prostate cancer. Urol J. (2016) 13:2527–32. doi: 10.22037/uj.v13i1.3077 26945657

[13] Zhu W Liu Y Hu K Li W Chen J Li J. Vitronectin correction of Vitronetcin promotes cell growth and inhibits apoptotic stimuli in a human hepatoma cell line via the activation of caspases. Can J Physiol Pharmacol. (2014) 92:363–8. doi: 10.1139/cjpp-2014-0032 24784470

[14] Carduner L Agniel R Kellouche S Picot CR Blanc-Fournier C Leroy-Dudal J. Ovarian cancer ascites-derived vitronectin and fibronectin: combined purification, molecular features and effects on cell response. Biochim Biophys Acta. (2013) 1830:4885–97. doi: 10.1016/j.bbagen.2013.06.023 23811340

[15] Zhu W Li W Yang G Fu C Jiang G Hu Q. Vitronectin silencing inhibits hepatocellular carcinoma. Vitro vivo. Future Oncol. (2015) 11:251–8. doi: 10.2217/fon.14.202 25179307

[16] Heyman L Leroy-Dudal J Fernandes J Seyer D Dutoit S Carreiras F. Mesothelial vitronectin stimulates migration of ovarian cancer cells. Cell Biol Int. (2010) 34:493–502. doi: 10.1042/CBI20090331 20121701

[17] Hao W Zhang X Xiu B Yang X Hu S Liu Z. Vitronectin: a promising breast cancer serum biomarker for early diagnosis of breast cancer in patients. Tumour Biol. (2016) 37:8909–16. doi: 10.1007/s13277-015-4750-y 26753956

[18] Bera A Subramanian M Karaian J Eklund M Radhakrishnan S Gana N. Functional role of vitronectin in breast cancer. PLoS One. (2020) 15:e0242141. doi: 10.1371/journal.pone.0242141 33211735 PMC7676670

[19] Keasey MP Jia C Pimentel LF Sante RR Lovins C Hagg T. Blood vitronectin is a major activator of LIF and IL-6 in the brain through integrin-FAK and uPAR signaling. J Cell Sci. (2018) 131:jcs202580. doi: 10.1242/jcs.202580 29222114 PMC5826040

[20] Nieberler M Reuning U Reichart F Notni J Wester H-J Schwaiger M. Exploring the role of RGD-recognizing integrins in cancer. Cancers (Basel). (2017) 9(9):116. doi: 10.3390/cancers9090116 28869579 PMC5615331

[21] Zheng DQ Woodard AS Tallini G Languino LR. Substrate specificity of alpha(v)beta(3) integrin-mediated cell migration and phosphatidylinositol 3-kinase/AKT pathway activation. J Biol Chem. (2000) 275:24565–74. doi: 10.1074/jbc.M002646200 10835423

[22] Karatug Kacar A Bolkent S. Vitronectin, fibronectin and epidermal growth factor induce proliferation via the JNK and ERK pathways in insulinoma INS-1 cells. Cytotechnology. (2019) 71:209–17. doi: 10.1007/s10616-018-0277-6 PMC6368526 30603922

[23] Chi M Liu J Mei C Shi Y Liu N Jiang X. TEAD4 functions as a prognostic biomarker and triggers EMT via PI3K/AKT pathway in bladder cancer. J Exp Clin Cancer Res. (2022) 41:175. doi: 10.1186/s13046-022-02377-3 35581606 PMC9112458

[24] Liang S Guo H Ma K Li X Wu D Wang Y. A PLCB1-PI3K-AKT signaling axis activates EMT to promote cholangiocarcinoma progression. Cancer Res. (2021) 81:5889–903. doi: 10.1158/0008-5472.CAN-21-1538 PMC9397629 34580062

[25] Sheng W Shi X Lin Y Tang J Jia C Cao R. Musashi2 promotes EGF-induced EMT in pancreatic cancer via ZEB1-ERK/MAPK signaling. J Exp Clin Cancer Res. (2020) 39:16. doi: 10.1186/s13046-020-1521-4 31952541 PMC6967093

[26] Wei R Xiao Y Song Y Yuan H Luo J Xu W. FAT4 regulates the EMT and autophagy in colorectal cancer cells in part via the PI3K-AKT signaling axis. J Exp Clin Cancer Res. (2019) 38:112. doi: 10.1186/s13046-019-1043-0 30832706 PMC6399964

